# Profiling of inflammatory mediators in the synovial fluid related to pain in knee osteoarthritis

**DOI:** 10.1186/s12891-020-3120-0

**Published:** 2020-02-14

**Authors:** Li Li, Zhenxing Li, Yuyan Li, Xi Hu, Yu Zhang, Pei Fan

**Affiliations:** 1 Zhejiang Provincial Key Laboratory of Anesthesiology, The Second Affiliated Hospital of Wenzhou Medical University, Yuying Children’s Hospital, Wenzhou, China; 2 Department of Anesthesiology, The Second Affiliated Hospital of Wenzhou Medical University, Yuying Children’s Hospital, Wenzhou, China; 3 Department of Orthopedics, The Second Affiliated Hospital of Wenzhou Medical University, Yuying Children’s Hospital, No.109, Xueyuan West Road, Wenzhou, China; 4 Zhejiang Provincial Key Laboratory of Orthopedics, The Second Affiliated Hospital of Wenzhou Medical University, Yuying Children’s Hospital, No.109, Xueyuan West Road, Wenzhou, China

**Keywords:** Pain, Osteoarthritis, knee, Inflammation mediators, Synovial fluid

## Abstract

**Background:**

Inflammatory mediators in the synovial fluid (SF) play critical roles in the initiation and development of pain in knee osteoarthritis (KOA). However, data for inflammatory marker expression are conflicting, and the role of SF inflammatory mediators in neuropathic pain is not clear. Therefore, the aim of this study was to identify SF inflammatory mediators associated with nociceptive and neuropathic pain in KOA.

**Methods:**

Levels of IL-1β, IL-6, TNF-α, macrophage colony-stimulating factor, MMP-3, MMP-13, metalloproteinase with thrombospondin motifs 5, calcitonin gene-related peptide, neuropeptide Y, substance P and bradykinin were measured using enzyme-linked immunosorbent assays in 86 patients. Nociceptive pain was assessed using the numeric rating scale (NRS), visual analog scale (VAS) and Western Ontario and McMaster Universities Osteoarthritis Index (WOMAC) pain score. Neuropathic pain was determined using the PainDETECT questionnaire. Moreover, knee function was evaluated by the WOMAC score and range of motion (ROM) assessments. Radiological grade was defined using the Kellgren-Lawrence (K-L) grading scale.

**Results:**

Pain scores measured using different methods correlated highly with each other. A worse K-L grade and knee function were associated with worse pain. Expression of IL-1β and IL-6 was increased in the early stage compared with the late stage. The NRS score correlated positively with age, K-L grade, and the WOMAC score and negatively with ROM and TNF-α expression. The VAS correlated positively with age, K-L grade, and the WOMAC score but negatively with ROM and levels of IL-1β, IL-6 and TNF-α. The WOMAC pain score did not correlate with any of the inflammatory mediators measured; it correlated only with ROM. The PainDETECT score correlated only with the WOMAC score. Expression of other inflammatory mediators did not correlate with any of the pain scores.

**Conclusions:**

IL-1β, IL-6 and TNF-α play critical roles in pain in the early stage of KOA and correlate with pain. The catabolic enzymes and neuropeptides measured do not correlate with nociceptive and neuropathic pain. New biomarkers related to pain in the late stage need to be further investigated.

## Background

Pain is the most prominent symptom of knee osteoarthritis (KOA) and the major driver of clinical decision-making. Despite great progress in the understanding of the molecular and cellular mechanisms of KOA, the treatment of pain is still a challenge in the clinic [1–4]. Therefore, etiological investigation of pain is not only helpful for understanding KOA but also critical for developing new medications to relieve it.

KOA was previously considered a “wear and tear” disease. However, in the past decade, inflammation has been found to play a critical role in the pathogenesis of KOA and to contribute to the initiation and development of pain [1, 5, 6]. Therefore, inflammatory mediators that serve as important factors that induce or reduce inflammation have attracted interest worldwide. Compared with inflammatory mediators in the serum, inflammatory mediators in the synovial fluid (SF) are thought to directly affect inflammation and cartilage metabolism and may constitute good targets for the treatment of pain.

Numerous types of SF inflammatory mediators, including inflammatory cytokines, matrix catabolic proteases and neuropeptides, have important functions. However, expression of these cytokines in osteoarthritis pain remains unresolved, and different studies have arrived at conflicting conclusions regarding interleukin 1β (IL-1β), interleukin 6 (IL-6) and tumor necrosis factor (TNF-α) [7–9]. In addition, the roles of some important inflammatory mediators, such as macrophage colony-stimulating factor (M-CSF) and metalloproteinase with thrombospondin motifs 5 (ADAMTS5), are still not clear. M-CSF was found to be required in the development of pain in a KOA experimental model; moreover, therapeutic neutralization of M-CSF reduced not only pain but also cartilage damage in patients with ankylosing spondylitis and rheumatoid arthritis [10, 11]. ADAMTS5 is an important enzyme involved in the degradation of the extracellular matrix (ECM) protein aggrecan [12], and ADAMTS5-deficient mice do not develop OA after resection of the medial meniscus [13]. Therefore, it is necessary to further explore the roles of these inflammatory mediators in pain and identify candidate inflammatory mediators associated with pain.

Furthermore, pain is classified as nociceptive or neuropathic [4], but few studies have investigated the correlation between SF inflammatory mediators and neuropathic pain. Therefore, another aim of this study was to examine correlations between neuropathic pain and SF inflammatory mediators.

## Methods

The protocol of this study was approved by the Ethics Committees of the Second Affiliated Hospital of Wenzhou Medical University (LCKY2019–174).

### Patient selection

Patients in our outpatient department diagnosed with primary KOA according to the American College of Rheumatology criteria were enrolled in the study [14]. Possible knee effusion was evaluated based on clinical symptoms and physical examination. The exclusion criteria included the following: 1. previous trauma; 2. symptoms of spinal disease; 3. prior treatment in the last 3 months; 4. joint replacement operation on the other knee; 5. cognitive disorders; 6. inflammatory arthritis (i.e., rheumatoid arthritis, spondylarthritis and gout); 7. fibromyalgia; 8. autoimmune disease (i.e., connective tissue disorders); and 9. septic arthritis.

### Patient characteristics and pain evaluation

Patient demographics and knee function parameters, including age, sex, range of motion (ROM) and the Western Ontario and McMaster Universities Osteoarthritis Index (WOMAC) score, were recorded [15]. The WOMAC score includes 3 subscales related to pain, stiffness and function; the higher is the WOMAC score, the worse is the joint function. The radiological grade of OA was evaluated according to the Kellgren-Lawrence (K-L) grading scale (0 = none, 1 = doubtful, 2 = minimal, 3 = moderate, and 4 = severe) [16]. In this study, K-L grades 1 and 2 were considered early-stage KOA; K-L grades 3 and 4 were considered as late-stage KOA. Nociceptive pain was evaluated using the visual analog scale (VAS) and numeric rating scale (NRS). The WOMAC pain score was employed to measure function-related pain, whereas neuropathic pain was measured according to the PainDETECT questionnaire (0–12 = unlikely, 13–18 = ambiguous, 19–38 = likely) [17].

### SF and an inflammatory mediator assay

SF was harvested with patient approval using a syringe and needle in the outpatient department during intra-articular injection. The aspirated SF was centrifuged at 3000 rpm for 10 min to remove cells and then stored at − 80 °C until use. Concentrations of inflammatory mediators were assessed using commercial enzyme-linked immunosorbent assay (ELISA) kits according to the manufacturer’s instructions (LanpaiBio, Shanghai, China). The inflammatory mediators measured included the following: inflammatory cytokines such as IL-1β, IL-6, TNF-α and M-CSF; matrix catabolic proteases such as matrix metalloproteinase 3 (MMP-3), matrix metalloproteinase 13 (MMP-13) and ADAMTS5; and neuropeptides such as calcitonin gene-related peptide (CGRP), neuropeptide Y (NPY), substance P (SP) and bradykinin (BK). Due to the low amounts of some samples, all cytokines were measured once.

### Statistical analysis

Data were statistically analyzed using SPSS 20.0 (IBM Corp., Armonk, NY, USA). Data with a normal distribution are reported as the mean and standard deviation. Data with a nonnormal distribution are reported as the mean and 95% confidence interval (CI). Correlation coefficients were determined by the Spearman rank correlation test using two-tailed *P* values. Comparison of values in different K-L groups was examined by analysis of variance (ANOVA) or the Kruskal-Wallis test depending on the results of the normality test and the Levene test. A correlation plot was generated using the R package (Version R 3.5.3, R Core Team, Vienna, Austria). A significant difference was considered at *p* < 0.05.

## Results

### Patient demographics, knee function and radiological assessment

**Fig. 1:**
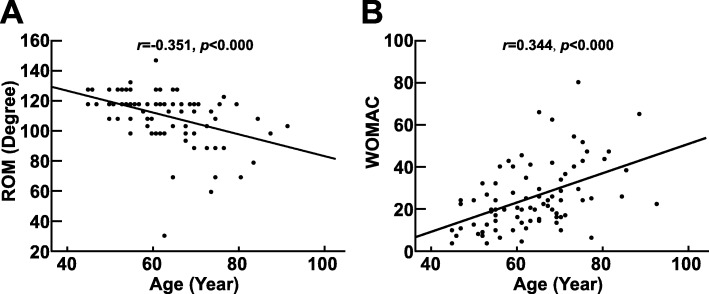
Correlation between age and knee function. **a**. Correlation between age and ROM. **b**. Correlation between age and WOMAC score. The correlation coefficient was calculated by the Spearman rank correlation test using two-tailed *p* values, as shown above the dot plots

**Fig. 2:**
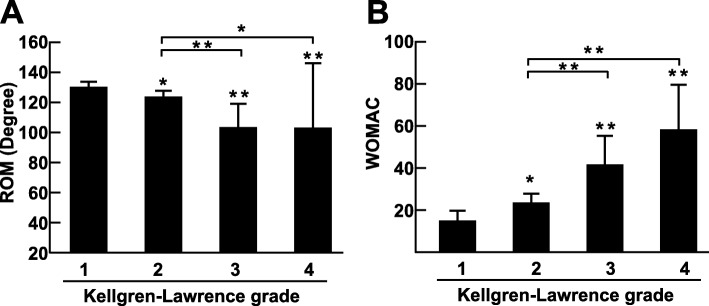
Knee function in different K-L groups. **a**. ROM in different K-L groups. Comparison of values was performed using the Kruskal-Wallis test. **b**. The WOMAC score in different K-L groups. Comparison of values was performed by one-way ANOVA. * *p* < 0.05 and ** *p* < 0.01

In total, 83 patients, including 21 males and 62 females, were enrolled in the study. The average age was 63.4 ± 10.4 years. The average WOMAC score was 25.5 (21.7–29.3); the average ROM was 112.0° (95% CI 108.0–116.1). The patient demographics, knee function, pain intensity and expression of inflammatory mediators in the different K-L grade groups are presented in Table 1. With increasing age, ROM became more limited (Fig. 1a), and the WOMAC score increased (Fig. 1b); moreover, worsening K-L grade led to limited ROM (*r* = − 0.499, *p* < 0.001) and an increased WOMAC score (*r* = 0.493, *p* < 0.001) (Fig. 2a and Fig. 2b), which indicated that knee function was gradually lost.

**Table 1:** Patient demographics, knee function, pain intensity and inflammatory mediator expression in different K-L grade groups

	K-L grade				Average
	1	2	3	4	
Male/Female	5/15	15/33	1/11	0/3	21/62
Age (years)	56.6 ± 9.2	63.3 ± 8.8	72.1 ± 11.2	74.0 ± 6.6	63.4 ± 10.4
ROM (°)	121.5 (117.7–125.2)	114.4 (110.1–118.6)	92.1 (75.1–109.1)	91.7 (44.6–138.7)	112.0 (108.0–116.1)
WOMAC	15.2 (10.6–19.7)	23.8 (19.7–27.9)	41.8 (28.2–55.3)	58.3 (37.2–79.5)	25.5 (21.7–29.3)
NRS	2.0 (1.4–2.5)	2.9 (2.5–3.3)	4.6 (3.3–5.9)	6.3 (−0.8–13.5)	3.04 (2.63–3.44)
VAS (cm)	2.5 (1.6–3.5)	3.5 (2.9–4.0)	4.7 (3.4–6.0)	7.0 (2.7–11.3)	3.55 (3.09–4.03)
WOMAC Pain	3.0 (2.0–4.0)	4.8 (4.0–5.6)	7.8 (4.7–10.8)	11.7 (5.9–17.4)	5.05 (4.29–5.81)
Neuropathic Pain	2.7 (0.9–4.5)	2.4 (1.5–3.4)	4.3 (1.5–7.0)	6.0 (−0.6–12.6)	2.89 (2.12–3.67)
IL-1β (pg/mL)	40.7 (37.4–44.0)	38.8 (36.8–40.9)	32.4 (28.7–36.2)	29.0 (5.40–52.7)	38.0 (36.3–39.6)
IL-6 (pg/mL)	30.3 (28.3–32.3)	29.7 (28.6–30.8)	26.7 (24.6–28.7)	25.9 (14.8–37.1)	29.3 (28.4–30.1)
TNF-α (pg/mL)	666.3 (591.2–741.5)	669.7 (616.6–722.8)	575.3 (473.2–677.4)	524.1 (64.4–1112.6)	650.0 (611.1–688.8)
M-CSF (pg/mL)	302.7 (273.7–331.8)	282.9 (266.2–299.6)	321.1 (293.8–348.4)	339.1 (321.6–356.6)	295.2 (282.7–307.8)
MMP-3 (ng/mL)	337.3 (351.4–403.2)	372.2 (355.2–389.2)	368.3 (328.0–408.6)	420.7 (321.8–519.6)	374.6 (362.0–387.2)
MMP-13 (ng/mL)	208.0 (192.8–233.2)	213.0 (200.6–225.4)	228.6 (219.7–246.5)	250.7 (146.9–354.5)	215.4 (206.9–224.0)
ADAMTS5 (U/mL)	45.3 (41.7–49.0)	42.6 (39.9–45.2)	44.6 (37.1–50.2)	41.5 (27.6–55.3)	43.5 (41.6–45.4)
BK (ng/mL)	5.67 (5.05–6.28)	5.68 (5.33–6.03)	6.15 (5.37–6.93)	6.28 (5.41–7.16)	5.77 (5.50–6.03)
CGRP (pg/mL)	94.7 (84.8–104.5)	91.4 (84.2–98.6)	95.1 (78.6–111.6)	109.9 (25.6–194.2)	93.4 (88.1–98.7)
SP (pg/mL)	109.7 (98.4–121.0)	105.6 (98.9–112.2)	103.3 (85.2–121.4)	96.2 (6.03–186.4)	105.9 (100.6–111.2)
NPY (ng/mL)	170.5 (155.2–185.8)	177.7 (168.9–186.4)	180.6 (161.9–199.2)	180.1 (111.3–249.0)	176.5 (169.9–183.1)

*ROM* range of motion; *NRS* numeric rating scale; *VAS* visual analog scale; *WOMAC* Western Ontario and McMaster Universities Osteoarthritis Index; *IL-1β* interleukin 1β; *IL-6* interleukin 6; *TNF-α* tumor necrosis factor α; *M-CSF* macrophage colony-stimulating factor; *MMP-3* matrix metalloproteinase 3; *MMP-13* matrix metalloproteinase 13; *ADAMTS5* metalloproteinase with thrombospondin motifs 5; *CGRP* calcitonin gene-related peptide; *NPY* neuropeptide Y; *SP* substance P; *BK* bradykinin

**Fig. 3:**
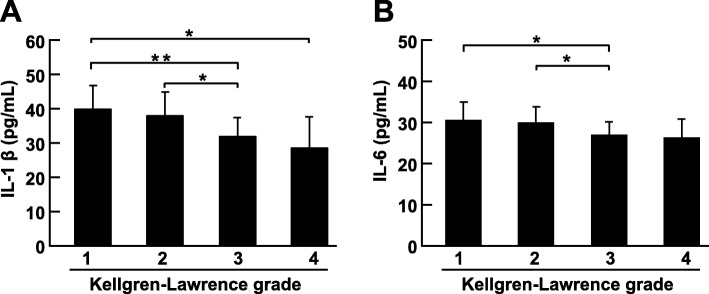
Expression of IL-1β and IL-6 in different K-L groups. **a**. Expression of IL-1β in different K-L groups. **b**. Expression of IL-6 in different K-L groups. Comparison of values was performed by one-way ANOVA. * *p* < 0.05 and ** *p* < 0.01

To explore differences in inflammatory mediators among different K-L groups, expression of inflammatory mediators was compared using one-way ANOVA. We found that IL-1β (*p* = 0.002) and IL-6 (*p* = 0.023) expression differed significantly among the four K-L groups (Fig. 3a and Fig. 3b). Increased expression of IL-1β and IL-6 was found in early-stage of KOA, which suggested that more severe inflammation may be present in KOA early stages. However, no significant differences were found for other inflammatory mediators among the K-L groups.

### Significant correlations among different pain scoring systems

To evaluate the intensity of pain in KOA, nociceptive pain was measured using the NRS and VAS, and neuropathic pain was determined using the PainDETECT questionnaire. The WOMAC pain score was recorded to measure function-related pain. NRS and VAS scores were 3.04 (95% CI 2.63–3.44) and 3.55 (95% CI 3.09–4.03) cm, respectively. The WOMAC pain score was 5.05 (95% CI 4.29–5.81); the PainDETECT pain score was 2.89 (95% CI 2.12–3.67).

**Fig. 4:**
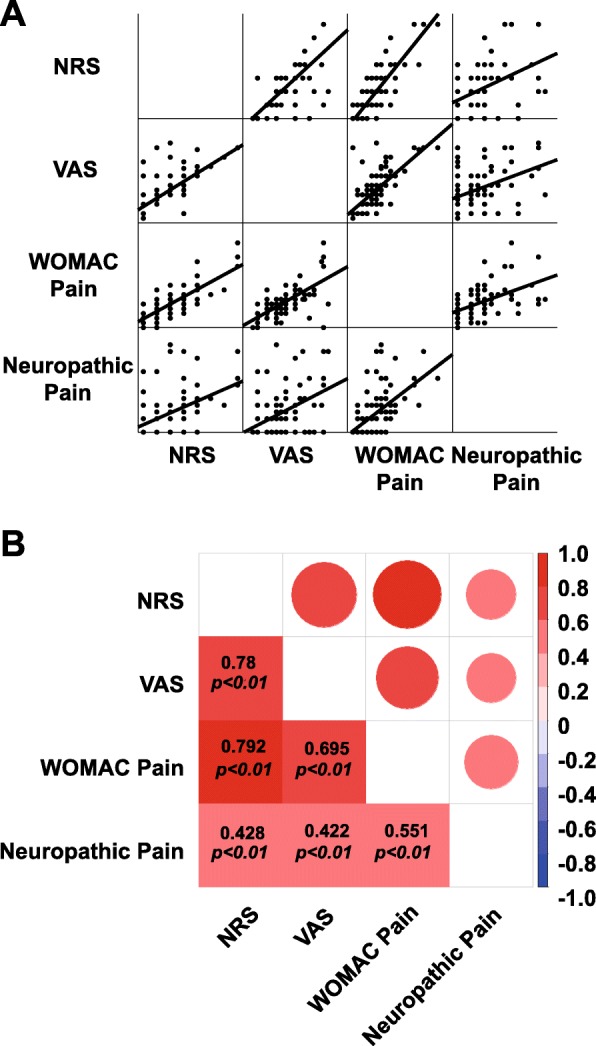
Pain scores measured using different methods and correlations among the methods. **a**. Pain scores measured using different methods. **b**. Correlation coefficients between different pain scoring systems. The different colors and circle areas represent the correlation coefficient between the pain scoring systems. Correlation coefficients were calculated by the Spearman rank correlation test using two-tailed *p* values

To further investigate consistency among the different pain scoring systems, correlation coefficients were determined using the Spearman test. As expected, the four measurements of pain correlated highly with each other and exhibited homogeneity (Fig. 4a and Fig. 4b).

### Pain affected knee function and correlated with age and K-L grade

**Fig. 5:**
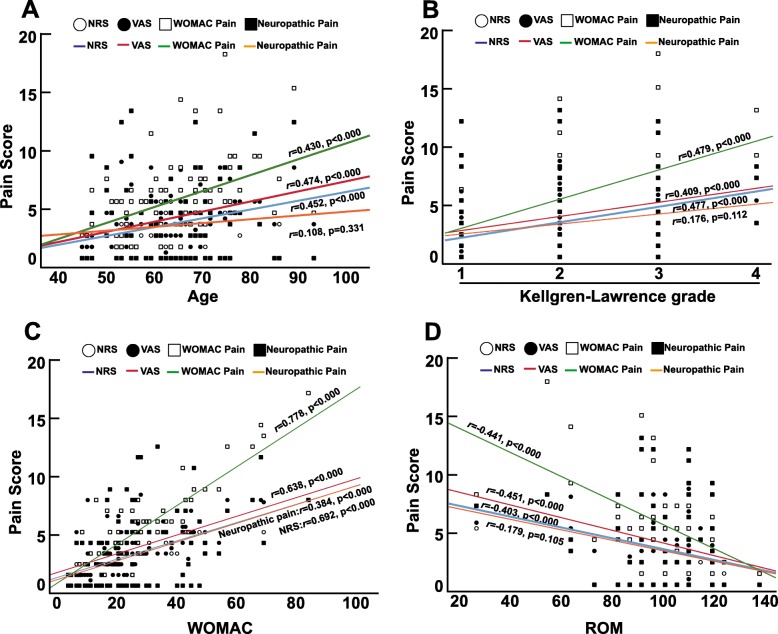
Correlations between pain and patient characteristics. **a**. Correlation between age and different pain scoring systems. **b**. Correlation between K-L grade and different pain scoring systems. **c**. Correlation between the WOMAC and different pain scoring systems. **d**. Correlation between ROM and different pain scoring systems. Correlation coefficients were calculated by the Spearman rank correlation test using two-tailed *p* values. Fitting curves with correlation coefficients are shown in the plots

To determine correlations between pain and patient demographic parameters, knee function or radiological grade, pain was analyzed for correlations with age, ROM, WOMAC score and K-L grade using the Spearman test. With an increase in age or K-L grade, NRS, VAS and WOMAC pain scores also increased, as expected (Fig. 5a and Fig. 5b). Correspondingly, knee function was gradually lost, as shown by a rise in the WOMAC score (Fig. 5c) and limited ROM (Fig. 5d). Nonetheless, neuropathic pain scores correlated only with the WOMAC score (*r* = 0.384, *p* < 0.000) and not age (*r* = 0.108, *p* = 0.331), K-L grade (*r* = 0.176, *p* = 0.112) or ROM (*r* = − 0.179, *p* = 0.105). In conclusion, age and K-L grade are important factors correlated with KOA pain, and KOA pain significantly affects knee function.

### Correlations among pain, patient characteristics and inflammatory cytokines

Inflammatory cytokines play critical roles in pain initiation and development [5]. Among inflammatory cytokines, IL-1β, IL-6 and TNF-α are the most important involved in KOA. Although IL-1β, IL-6 and TNF-α have been widely investigated in both experimental models and clinical samples, expression of these cytokines in KOA pain remains unresolved, and different research studies have reached conflicting conclusions [7, 18, 19]. Moreover, as mentioned in the introduction section, M-CSF has been found to be important in KOA [10, 11]. However, whether SF M-CSF is involved in KOA pain is still unknown.

To investigate correlations between pain and the above inflammatory cytokines, expression of IL-1β, IL-6, TNF-α and M-CSF was measured by ELISA, and the results were analyzed by the Spearman test. As shown in Table 2, IL-1β and IL-6 very weakly correlated negatively with the VAS score; TNF-α correlated negatively with both VAS and NRS scores. In contrast, M-CSF did not show any correlation with any of the pain scores. Moreover, WOMAC-assessed pain and neuropathic pain did not correlate with any of the inflammatory cytokines. In short, the above evidence demonstrates that inflammatory cytokines correlate significantly with KOA pain. In general, expression of IL-1β, IL-6 and TNF-α may be higher in patients with less pain.

**Table 2:** Correlations between inflammatory cytokines and pain scoring systems

	IL-1β	IL-6	TNF-α	M-CSF
VAS	−0.280*	− 0.272*	− 0.249*	0.018
NRS	−0.203	− 0.186	− 0.270*	−0.019
WOMAC Pain	0.185	−0.139	−0.186	− 0.044
Neuropathic Pain	−0.019	− 0.034	−0.115	− 0.001

*NRS* numeric rating scale; *VAS* visual analog scale; *WOMAC* Western Ontario and McMaster Universities Osteoarthritis Index; *IL-1β* interleukin 1β; *IL-6* interleukin 6; *TNF-α* tumor necrosis factor α; *M-CSF* macrophage colony-stimulating factor. * *p* < 0.05 ** *p* < 0.01.

To further determine correlations between inflammatory cytokine expression and patient characteristics, expression of IL-1β, IL-6, TNF-α and M-CSF was analyzed for correlations with age, K-L grade, ROM and WOMAC score using the Spearman test (Table 3). IL-1β and IL-6 correlated negatively with age, K-L grade and WOMAC score, whereas IL-6 correlated positively with ROM. In addition, TNF-α correlated negatively with age. In line with the results illustrated in Fig. 3, expression of IL-1 and IL-6 was relatively high in the early stage compared with the late stage of KOA, and expression of these two inflammatory cytokines was associated with knee function.

**Table 3:** Correlations between inflammatory cytokines and patient characteristics

	Age	K-L grade	ROM	WOMAC
IL-1β	−0.272*	− 0.363**	0.214	− 0.317**
IL-6	−0.261*	− 0.291**	0.219*	− 0.297**
TNF-α	−0.240*	− 0.149	−0.033	− 0.119
M-CSF	0.085	0.123	−0.033	−1.00

*IL-1β* interleukin 1β; *IL-6* interleukin 6; *TNF-α* tumor necrosis factor α; *M-CSF* macrophage colony-stimulating factor; *K-L grade* Kellgren-Lawrence grade; *ROM* range of motion; *WOMAC* Western Ontario and McMaster Universities Osteoarthritis Index. * *p* < 0.05 ** *p* < 0.01.

### No correlations were found between pain and measured catabolic proteases

Progression of OA is usually accompanied by high matrix catabolism, we hypothesized that high expression of catabolic proteases in the SF promotes the breakdown of cartilage and causes severe synovitis and inflammation, which may lead to increased pain. Among these catabolic proteases, MMPs and ADAMTS5 play important roles in KOA through involvement in the degradation of ECM type II collagen and aggrecan, respectively [20, 21]. However, it remains unknown whether these proteases are involved in KOA pain.

To evaluate correlations between these catabolic proteases and pain, MMP-3, MMP-13 and ADAMTS5 levels were measured by ELISA, and the results were correlated with pain scores using the Spearman test (Additional file 1: Table S1). According to the results, only MMP-13 showed a correlation, albeit weak, with the NRS score (*r* = 0.267, *p* = 0.015). No correlations were found between pain scores and the other measured catabolic enzymes. This result indicates that the catabolic cytokines measured may not be involved in KOA pain.

### No correlations were found between pain and measured neuropeptides

It is believed that neuropeptides sensitize knee nociceptors and are related to knee pain [19], and SP, NPY, CGRP and BK are considered important neuropeptides involved in OA pain. First, SP in the SF has been reported in KOA and rheumatoid arthritis [22], and the concentration of SP is significantly higher in painful temporomandibular joints than in painless temporomandibular joints [23]. Second, expression of NPY in the dorsal root ganglion was found to correlate significantly with OA pain in animal models [24], and SF NPY expression correlates with OA pain [25]. Third, CGRP is reportedly involved in the progression and prognosis of KOA and widely distributed in nociceptive pathways in the peripheral and central nervous systems, correlating with numerous types of pain [26–28]. Lastly, BK is an inflammatory mediator that can lead to vasodilation and inflammation induction. Although BK has been reported to correlate with biomarkers for cartilage degradation and inflammation in OA [29], the role of BK in pain needs to be further investigated. Therefore, it is necessary to explore whether these neuropeptides play roles in OA pain.

To address this issue, expression of these neuropeptides was analyzed, and the Spearman test was performed to examine correlations with different pain scoring systems (Additional file 2: Table S2). No correlations were found between any of the measured pain scoring systems and these neuropeptides, which suggests that these neuropeptides may not contribute to KOA pain.

## Discussion

Pain is the major symptom of KOA and needs to be further investigated. Although complicated mechanisms are involved in OA pain, our study focused only on SF inflammatory mediators because we think that increasing or reducing the levels of certain inflammatory mediators may shed new light on pain treatment.

Unlike the structural changes occurring in OA, the greatest obstacle in pain research is the subjective nature of the field [3, 30]. To minimize subjective effects, pain intensity was measured in this study using four different pain scoring systems: VAS, NRS, WOMAC pain score and PainDETECT score. The VAS and NRS are commonly used pain measurement tools. Although the VAS is frequently used in pain research, it is more complicated than the NRS, especially when used for elderly patients due to cognitive impairments [31]. For this reason, we decided to apply both pain scoring systems to evaluate pain intensity. A previous study reported correlations between the VAS and NRS ranging from 0.62–0.91 [32]. In line with this result, our correlation coefficient was 0.775 (*p* < 0.000). Moreover, the WOMAC pain score is widely utilized in KOA pain research because it is more precise than the VAS and NRS and can reflect pain at rest and during movement [33]. In addition, it has been demonstrated that pain can be classified as nociceptive and neuropathic pain [4]. For this purpose, we also evaluated neuropathic pain using the PainDETECT questionnaire. To our knowledge, few studies have focused on the correlation between neuropathic pain and nociceptive pain. In our study, we found that neuropathic pain scores had a weak correlation with the NRS (*r* = 0.428, *p* < 0.000), VAS (*r* = 0.422, *p* < 0.000) and WOMAC pain score (*r* = 0.551, *p* < 0.000). Therefore, our results indicate that all of these pain measurement methods have high internal consistency and are reliable measures to be used in research.

Patient characteristics correlate with pain in OA [30]. First, old age is thought to be a systemic risk factor for OA [34, 35], and it correlates with pain [36]. In our study, we found that age correlated significantly with pain scores (*r* = 0.430–0.474, *p* < 0.000) and played an important role in the genesis of OA. Second, radiological images are important tools for evaluating OA severity and predicting knee pain. A positive correlation between knee pain and K-L grade has been found in several studies [37, 38]. For example, McAlindon reported a correlation coefficient of 0.43. In line with this result, our study suggested that K-L grade correlated significantly with pain scores (*r* = 0.409~0.479, *p* < 0.000) but not with neuropathic pain scores (*r* = 0.176, *p* = 0.112). Third, pain significantly affects ROM, as demonstrated by numerous studies [36, 37]. In agreement with previous reports, we found that patient ROM correlated highly with pain scores (*r* = − 0.403~ − 0.451, *p* < 0.000). In summary, as age increases and radiological assessments show worsening results, patient pain increases, and joint function is lost.

It is well known that inflammatory cytokines play critical roles in OA pain. Although M-CSF is a very important inflammatory cytokine, to our knowledge, no previous research has focused on the correlation between SF M-CSF and pain intensity. Therefore, we hypothesized that an increased SF M-CSF level would lead to more severe pain because M-CSF induces inflammation. However, no significant correlation was found between pain and the concentration of M-CSF in this study. Accordingly, the strategy of reducing M-CSF expression may not be effective for alleviating OA pain. Overall, the roles of some common SF inflammatory cytokines, such as IL-1β, IL-6 and TNF-α, are still controversial. Radojcic reported that IL-6 in the SF is significantly positively associated with knee pain based on the WOMAC pain score (B = 0.022; 95% CI 0.004–0.040) [8]. In contrast, Brenner reported that IL-6 levels have no correlation with the WOMAC pain score [7]. Orita reported that TNF-α is positively related to pain but that IL-6 is not [9]. Thus, different research studies have obtained conflicting results. This discrepancy may be due to the subjective nature of pain and might be influenced by disease stage. In this study, we found that IL-1β and IL-6 correlated negatively with K-L grade and pain scores. We hypothesized that in the early stage of KOA, inflammation in the knee joint may be the major reason that patients seek help in an outpatient department; in the late stage, pain may not derive from inflammation but rather from other sources that need to be further investigated. This finding is consistent with the results of previous reports. Barker found that in early-stage KOA, serum levels of TNF-α and IL-6 were significantly higher than during the late stage [39]. Orita also reported that IL-6 in the SF has a relatively significant negative correlation with K-L grade but that TNF-α does not [9]. Additionally, Ene found that compared with those in the late stage of OA, expression of inflammatory mediators and infiltration of mononuclear cells in the early stage were enhanced [40]. Combining these results and ours, we suggest that anti-inflammatory treatment may be more useful in the early stage than in the late stage and that a new treatment strategy for controlling pain should be considered for the late stage of KOA.

High catabolism and high anabolism coexist in OA patients, but the degree of former is more extensive than that of latter [41]. In addition, it is important to evaluate catabolism-related proteinases to monitor the progression of OA. Therefore, we hypothesized that high expression of catabolic proteinases may result in matrix fragmentation, which might induce inflammation and cause more pain. A previous study showed that MMP-3, MMP-13 and ADAMTS5 in the SF can be detected in KOA [42, 43]. Although ADAMTS5 plays a critical role in degrading aggrecan, to our knowledge, this is the first report to explore the correlation between ADAMTS5 and pain. Regardless, after comparing ADAMTS5 with various pain scoring systems, no correlation was found between ADAMTS5 and pain in this study. In addition, only expression of MMP-13 had a weak correlation with the NRS. These results are similar to previous results. Bay-Jensen reported that serum COL3/ADAMTS was weakly associated with pain scores (*r* = − 0.13–0.17, *p* < 0.05) [44]. Sun compared MMP-3 and NRS but did not find any correlation [45]. Moreover, no other associations between SF catabolic proteases and pain have been found, except for the above-mentioned results. For this reason, we conclude that the catabolic proteases measured may not be involved in KOA pain and that more catabolic proteases should be analyzed in the future.

Neuropeptides have been reported to be involved in OA pain in many experimental models and clinical patients [3, 19], but whether these neuropeptides are related to pain is still unclear. Hence, we measured expression of these neuropeptides and attempted to identify correlations with pain. First, NPY is an important neuropeptide involved in pain occurrence. Wang et al. reported that NPY in the SF is related to OA pain [25], though we did not find any correlations after comparing four types of pain scoring systems with NPY expression. This disagreement may be due to the use of Hideo Watanabe’s pain score, which only includes 5 levels of pain and is not as accurate as the VAS, NRS or WOMAC pain score, in the study by Wang et al.; in addition, correlation coefficients for different pain subgroups were not available in their research. Therefore, the role of NPY needs to be further investigated. Second, SF SP has been reported in KOA patients [22], and the level of SP has been correlated with pain relief after treatment with medication [46]. The SP receptor (TACR1) gene has also been correlated with pain in KOA [47], though to date, no study has reported a link between SP and pain intensity. Our study showed no correlation between SF SP and OA pain. Third, CGRP is an important neuropeptide produced in both peripheral and central neurons that is involved in numerous types of pain, such as headache [26]. It has been reported that increasing CGRP expression in the SF contributes to the progression of arthritis in developmental dysplasia of the hip [48]. Takano reported that elevation of CGRP levels in the synovium may contribute to OA pain [49], and Dong reported that CGRP correlates with KOA pain using the WOMAC pain score (*r* = 0.524, *p* < 0.001) [27]. Conversely, we did not observe a correlation between CGRP and pain intensity. Lastly, BK is an inflammatory mediator that can lead to vasodilation and inflammation induction, and SF levels of BK have been reported to correlate with biochemical markers of cartilage degradation and inflammation in KOA [29]. In our study, BK did not correlate with any pain score, a result that is consistent with previous findings. After evaluating correlations between BK and pain in OA, Bellucci did not observe any correlation [29]. In short, the neuropeptides measured did not correlate with OA pain in our study.

The present study has some limitations. First, the number of patients with an advanced K-L grade was limited. This limitation prevented us from further exploring the factors that influence pain in the late stage of KOA. Second, because patients likely having neuropathic pain were defined as a PainDETECT score higher than 18, we did not identify any patients likely to have neuropathic pain in this study. This limitation indicates that we need to further explore the risk factors for neuropathic pain.

Pain is the major symptom of KOA and needs to be further investigated. Because pain is divided into nociceptive and neuropathic pain, we suggest that future studies use measurements that represent nociceptive and neuropathic pain to explore the mechanisms. In addition, to better illustrate the association between SF inflammatory mediators and OA-related pain, randomized and long-term clinical trials that control for demography, K-L grade and knee function are needed to understand the initiation and development of KOA pain and are essential for the development of pain-targeted medication.

## Conclusion

IL-1β, IL-6 and TNF-α exhibit higher expression in the early stage than in the late stage of KOA and correlated with pain, indicating that anti-inflammation may be more efficient in alleviating pain in the early stage. The catabolic enzymes, including MMP-3, MMP-13 and ADAMTS5, and neuropeptides, including CGRP, NPY, SP and BK, measured do not correlate with nociceptive or neuropathic pain. New biomarkers related to pain in the late stage need to be further investigated.

## Supplementary information

**Additional file 1: Table S1.** Correlations between catabolic cytokines and pain.

**Additional file 2: Table S2.** Correlations between neuropeptides and pain.

## Data Availability

The datasets used and analyzed during the current study are available from the corresponding author on reasonable request.

## Abbreviations

ADAMTS5: Metalloproteinase with thrombospondin motifs 5
ANOVA: Analysis of variance
BK: Bradykinin
CGRP: Calcitonin gene-related peptide
CI: Confidence interval
ECM: Extracellular matrix
ELISA: Enzyme-linked immunosorbent assay
IL-1β: Interleukin 1β
IL-6: Interleukin 6
K-L grading scale: Kellgren-Lawrence grading scale
KOA: Knee osteoarthritis
M-CSF: Macrophage colony-stimulating factor
MMP-13: Matrix metalloproteinase 13
MMP-3: Matrix metalloproteinase 3
NPY: Neuropeptide Y
NRS: Numeric rating scale
ROM: Range of motion
SF: Synovial fluid
SP: Substance P
TNF-α: Tumor necrosis factor
VAS: Visual analog scale
WOMAC: Western Ontario and McMaster Universities Osteoarthritis Index score
