# Supplementary material for: Profiling of inflammatory mediators in the synovial fluid related to pain in knee osteoarthritis
Source: BMC Musculoskelet Disord. 2020 Feb 14;21:99. doi: 10.1186/s12891-020-3120-0 (PMC7023718; doi:10.1186/s12891-020-3120-0)
Supplement: Supplementary file 1 — Additional file 1: Table S1. Correlations between catabolic cytokines and pain. [file 12891_2020_3120_MOESM1_ESM.docx]

Supplement Table 1. Correlations between catabolic cytokines and pain.

|  | **MMP-3** | **MMP-13** | **ADAMTS5** |
| --- | --- | --- | --- |
| **VAS** | 0.000 | 0.216 | -0.031 |
| **NRS** | 0.079 | 0.267* | 0.041 |
| **WOMAC Pain** | 0.205 | 0.102 | -0.056 |
| **Neuropathic Pain** | 0.175 | 0.209 | 0.180 |

**NRS**: numeric rating scale, **VAS**: visual analog scale, **WOMAC**: Western Ontario and McMaster Universities Osteoarthritis Index, **MMP-3**: matrix metalloproteinase 3, **MMP-13**: matrix metalloproteinase 13, **ADAMTS5**: metalloproteinase with thrombospondin motifs 5. * *p*<0.05 ** *p*<0.01
