# Supplementary material for: Profiling of inflammatory mediators in the synovial fluid related to pain in knee osteoarthritis
Source: BMC Musculoskelet Disord. 2020 Feb 14;21:99. doi: 10.1186/s12891-020-3120-0 (PMC7023718; doi:10.1186/s12891-020-3120-0)
Supplement: Supplementary file 2 — Additional file 2: Table S2. Correlations between neuropeptides and pain. [file 12891_2020_3120_MOESM2_ESM.docx]

Supplement Table 2. Correlations between neuropeptides and pain.

|  | **SP** | **NPY** | **CGRP** | **BK** |
| --- | --- | --- | --- | --- |
| **NRS** | -0.163 | 0.018 | 0.070 | 0.051 |
| **VAS** | -0.103 | -0.011 | -0.089 | -0.064 |
| **WOMAC Pain** | -0.008 | -0.150 | -0.149 | -0.013 |
| **Neuropathic Pain** | 0.007 | -0.173 | -0.131 | 0.019 |

**NRS**: numeric rating scale, **VAS**: visual analog scale, **WOMAC**: Western Ontario and McMaster Universities Osteoarthritis Index, **CGRP**: calcitonin gene-related peptide, **NPY**: neuropeptide Y, **SP**: substance P, **BK**: bradykinin. * *p*<0.05 ** *p*<0.01
